# Quaternion Model of Higher-Order Rotating Polarization Wave Modulation for High Data Rate M2M LPWAN Communication

**DOI:** 10.3390/s21020383

**Published:** 2021-01-07

**Authors:** Zaid Ahmad, Shaiful Jahari Hashim, Fakhrul Zaman Rokhani, Syed Abul Rahman Al-Haddad, Aduwati Sali, Ken Takei

**Affiliations:** 1 WiPNET Research Center, Department of Computer and Communication Systems Engineering, Universiti Putra Malaysia, Serdang, Selangor 43400, Malaysia; fzr@upm.edu.my (F.Z.R.); sar@upm.edu.my (S.A.R.A.-H.); aduwati@upm.edu.my (A.S.); 2 Department of Electrical and Computer Engineering, COMSATS University (CUI), Lahore 54600, Pakistan; 3 Research and Development Group, Hitachi Ltd., Tokyo 185-8601, Japan; ken.takei.cb@hitachi.com

**Keywords:** polarization diversity, LPWAN, machine-to-machine communication, phase-shift keying, industrial communication systems, sub-gigahertz band

## Abstract

With growing interest in Industry 4.0, machine-to-machine communication (M2M) will become the key enabler for low-power wide area networks (LPWANs) in connecting machines and sensor nodes distributed across a distance in the industrial environment. The choice of modulation and diversity techniques, and the selection of spectrum (licensed/unlicensed) will impact and influence the requirements of wireless M2M systems. Link reliability is one of the most important requirements for LPWAN deployment in industrial scenarios. Rotating Polarization Wave (RPW) system has been recently proposed as an LPWAN solution for reliable M2M communication in high clutter environment and it deploys BPSK modulation with polarization diversity (PD). This paper proposes a new multi-level Rotating Polarization Phase-Shift Keying (RP-MPSK) modulation to provide high data rate and energy efficiency. A novel quaternion model for RPW system (Q-RPW) is also proposed to reduce the complexity in modeling, simulation, and implementation. Results using Q-RPW model show that RP-MPSK modulation offers a high diversity gain over BPSK with second-order diversity. Bit error rate (BER) performance of RP-MPSK modulation compared against other LPWAN modulation like MPSK, FSK and QAM has shown high reliability and substantial improvement in SNR. To overcome the degradation in error performance caused by the proposed higher-order modulation, sampling rates are recommended based on BER performance. BER performance of RP-MPSK under multipath and interference conditions is also investigated.

## 1. Introduction

The emergence of LPWANs shall transform Industry 4.0 into a reality in the upcoming years. M2M communication is believed to be the key enabler for this transformation. The concept of M2M has evolved to Internet of things (IoT) after increasing attention from scholars and practitioners [1]. The penetration of IoT into industry, termed as Industrial Internet of Things (IIoT), has revolutionized the paradigm of industrial communication systems (ICSs) by coping with stringent requirements [2]. From a wider perspective, ICSs are supposed to provide seamless access to the network segments or single nodes placed at the lowest level of an industrial automation system. The most important requirements are timeliness, reliability, and flexibility. The timeliness implies that the system must be capable of carrying out communication tasks to deliver a message within the stipulated time [2]. Reliability is another strict requirement for industrial scenarios. Link reliability can be achieved by spatial and temporal redundancies [3]. The flexibility includes scalability, reconfiguration, and reassembly [2,4].

ICSs can be classified into critical and non-critical systems [5]. The critical systems are specified by high reliability and data rates that enable real-time communication, while moderate data rates are acceptable for non-critical systems. ICSs mainly comprise fixed transmitters and receivers as the industrial assets are least likely to move. However, establishing a reliable communication is highly challenging because of heavily obstructive environment. Robustness and data rate are therefore primary design requirements in critical systems. These design goals greatly influence the design decisions such as modulation techniques, diversity techniques, and spectrum (licensed/unlicensed) to be used. Studies have been conducted to investigate the suitability of various LPWAN technologies to enable industrial communication [4,5,6]. B. Buurman et al. [5] and N. Xia et al. [7] recommend NB-IoT for long range critical systems while LoRa for medium range. A. Seferagić et al. [8] consider LoRa, Wi-Fi HaLow and NB-IoT as the most suitable technologies over a range comparable to industrial site. The reliability mechanism of LoRa is orthogonal spreading factors (SF), while Wi-Fi HaLow and NB-IoT use forward error correction (FEC). LoRa data rate is 50 kbps which is the lowest of the recommended technologies. Wi-Fi HaLow outstands with a minimum data rate of 150 kbps while NB-IoT offers a data rate that does not exceed 125 kbps. In addition to the minimum data rate, LoRa compromises the link reliability in a harsh environment with elevated perturbations. Experiments carried out by K. Staniec and M. Kowal [9] in the reverberation and anechoic chambers with SF of 7–9 have shown 100% packet error rate (PER). LoRa shows a better resilience to multipath only with SF from 10–12 but higher spreading means lower data transmission rate. A simulation study on the use of LoRa in underground mines has reported a 2.5 dB to 6 dB performance reduction to achieve a bit error rate (BER) as low as $10^{-3}$ [10]. NB-IoT and Wi-Fi HaLow offer high reliability, but they have limitations on data rate and range, respectively. As mentioned earlier, the maximum data rate of NB-IoT is 125 kbps while the maximum range covered by Wi-Fi HaLow is 1 km [11]. Therefore, certain trade-off decisions are involved in selecting a suitable LPWAN technology for an industrial scenario.

Modulation technique is an important design decision in deployment of the physical layer (PHY) of an ICS [5,6]. Complex modulation schemes can cause an increase in power consumption of transmitter and receiver. Modulation technique also affects latency [4], latency variation (jitter) and communication range [8]. Modulations with fewer constellation points are more reliable but they are slower at the same time. Higher data rates can be achieved by encoding more data into the physical signal. Phase-Shift Keying (PSK) and Quadrature Amplitude Modulation (QAM) are the most popular modulation techniques in LPWANs [5]. Higher-order PSK and QAM modulations increase data rate and spectral efficiency, with a compromise on bit error rate (BER). However, QAM is 400% less power efficient than PSK. LoRa uses Chirp Spread Spectrum (CSS) and Frequency-Shift Keying (FSK); NB-IoT uses binary phase-shift keying (BPSK) and quaternary phase-shift keying (QPSK) while Wi-Fi HaLow employs BPSK, QPSK and higher order QAM modulations [11]. If the error performance can be made consistent with the requirement, PSK is preferred over QAM. This can be achieved if a suitable diversity method is used with PSK modulation.

Diversity techniques improve link reliability of LPWANs. The situations in which diversity helps include networks in remote areas, sparse location of nodes and hostile scattering environments [5]. They have moderate to high impact on overall power consumption, range, cost, and interference management of the system. Diversity methods can be broadly categorized into frequency, time, and spatial diversity. One of the design goals of the LPWANs is power and spectrum efficiency. Industrial entities are shifting to unlicensed spectrum that offers small bandwidth but with cheaper overall deployment and maintenance cost. Therefore, frequency diversity alone cannot offer desired redundancy. Time diversity is a better approach, but it is not power efficient because of retransmission [5]. Spatial diversity (SD) is the predominant diversity method with the rise of multiple-input multiple-output (MIMO) systems but not suitable for the lower frequency industrial, scientific, and medical (ISM) frequency bands. The size of the MIMO antenna structure can be prohibitive and increase the power consumption considerably. Specifically, SD based on MIMO requires large antenna spacing to maintain orthogonality. Thus, the antennas at the base station should be distanced tens of wavelengths apart, while separation comparable to a wavelength at mobile station is required [12]. However, using multiple diversity techniques together can optimize the performance of communication link. Polarization diversity (PD) is regarded as a spatial efficient and effective alternative to SD in Non-Line-of-Sight (NLoS) environment because the signals transmitted through two orthogonal polarizations are independent [13]. F. Challita et al. [14] have investigated the performance of PD for a massive MIMO system in industrial environment to address the problem of cross-polarization discrimination. F. Challita et al. [15] have reported significant improvement in spectral efficiency in PD based massive MIMO system for Industry 4.0 applications. There is a gap, however, in existing literature on exploring PD for M2M and industrial communication. PD should be further investigated to improve link reliability in industrial scenarios.

Several channel models have been proposed for PD systems [16]. They can be classified as physical or analytical models. The physical models are mostly developed using the exact ray-tracing or the geometric approach. However, they do not offer flexibility to design polarization-based technologies for more general scenarios. Analytical models on the other hand allow for mathematical representation of dual-polarized systems. Most of the models belonging to this class are based on correlation that characterize the channel matrix statistically. Analytical models are more suitable for Rayleigh fading channels that correspond to NLoS scenarios. However, they do not account for actual propagation effects such as scattering and channel depolarization. The models that incorporate these effects are cumbersome and lack analytical tractability. To solve these problems, Wysocki B. et al. [17] have proposed a quaternion-based model for dual-polarized channel. The model offers a way to differentiate the two orthogonal polarizations and generate fading channels with cross-polar scattering and channel depolarization. Another significant advantage of the model is the reduction in complexity involved in classical channel modelling by halving the required number of real random variables [17]. The method has also been used to optimize PD gain with dual-polarized antennas [18]. However, the model has not been used to evaluate the performance of M2M communication.

**Figure 1:**
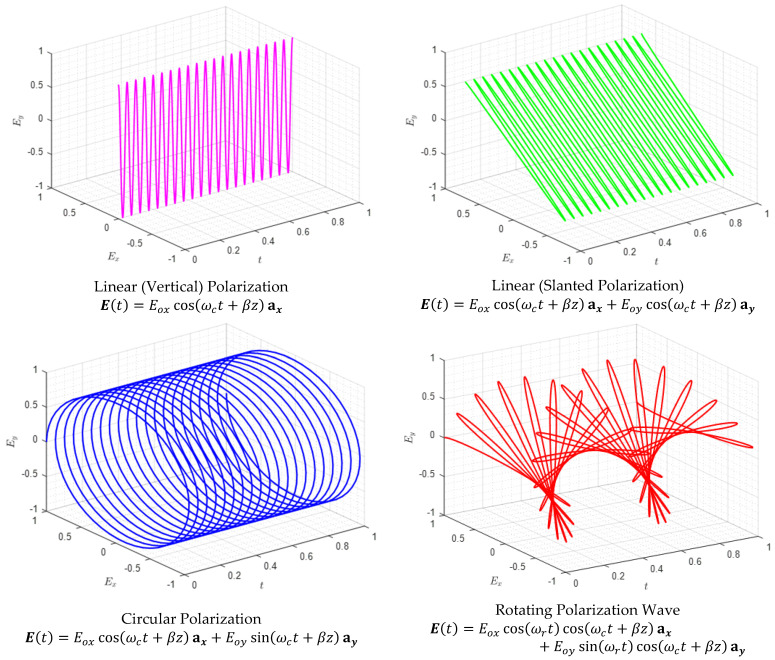
Comparison of RPW with conventional forms of polarization.

Rotating Polarization Wave (RPW) is a newly emerging LPWAN that provides highly reliable M2M communication. Since RPW is a nascent technology, it has not been widely discussed in literature. A prototype was developed, and its performance was experimentally evaluated [19]. However, there is no commercial module available. Providing deterministic communication in a highly disruptive environment is the hallmark of the RPW communication. When transmitters and receivers are fixed as in a typical industrial environment, the power of regular reflected waves is significantly higher than the irregular reflected waves even if there is no Line-of-Sight (LoS) available between the transmitter and the receiver. These waves can be handled as a single direct wave in classical mobile radio environment [20]. The received signal polarization can be adaptively controlled by slowly rotating the polarization of transmitted signal, such that the message can be received at an arbitrary number of polarization angles. Theoretically, the received RPW signal is 10 dB stronger compared to the uni-polarized signal. The field tests in industrial environment conducted by K. Takei [20] have demonstrated higher received signal strength (RSS) and improved error performance. The existing method of RPW employs BPSK modulation that offers high reliability with a limited available bandwidth. Because of simple demodulation and minimum channel estimation requirement, its performance is superior to other modulation schemes in real-time applications. Since RPW is a PD technique, compact and power efficient base stations and mobile stations capable of RPW communication can be realized. Therefore, RPW communication is an attractive unification of efficient modulation and diversity techniques. Another useful feature of RPW is combining the strengths of PD and circular polarized (CP) systems. PD systems are often adopted in multipath environment because of their simple transmit and receive antenna structures. CP systems perform better than linear-polarized (LP) systems in cluttered propagation environment [13]. Such environments are formed by several flat surfaces like buildings, internal walls, and metallic structures. Another advantage of CP is a low delay-spread, that can mitigate jitter or delay variation in the received signal. RPW signal physically resembles the LP signal at carrier frequency, $\omega_{c}$; however, this LP signal is rotated at an angular frequency $\omega_{r}$ that is much lower than $\omega_{c}$. In fact, RPW is generated by transmitting two baseband signals of frequency $\omega_{r}$ having a phase difference of π/2 through a dual linear polarized antenna operating on ISM carrier frequency $\omega_{c}$. Hence RPW combines the spatial characteristics of CP and PD to offer an increased link reliability for a highly reliable M2M communication. Figure 1 shows a comparison between RPW and other forms of polarization at carrier frequency. For illustrative purpose, $\omega_{c}=40\pi$ rad/s, while $\omega_{r}$ for RPW is taken to be as low as 2π rad/s. The horizontal and vertical polarizations are denoted by $E_{x}$ and $E_{y}$ with amplitudes $E_{ox}$ and $E_{oy}$ respectively.

Existing prototype of RPW transceiver operates on Sub-Gigahertz ISM band and offers a data rate of 125 kbps using RP-BPSK modulation [20] which is comparable to NB-IoT and more than double the data rate of LoRa. However, this data rate is much lower that the data rate of Wi-Fi HaLow mentioned above. RPW can overcome this data rate limitation if higher order modulations are being used. Data rate is increased because a greater number of bits are transmitted within the same symbol period. This translates to spectral efficiency because the same bandwidth is used for more bits per second. The data rate improvement is also necessary because the performance of LPWANs is limited by duty cycle regulations. The duty cycle percentage available for a sensor node can be more efficiently used if more data can be transmitted during that time.

In case of LPWANs and sensor networks, active transmission is the most power intensive mode [21,22]. Use of higher order modulations can also improve energy efficiency, because in higher order modulation, the same symbol energy is used to encapsulate more amount of information than a lower order modulation [23]. However, these improvements come at the cost of higher BER. This degradation in error performance can be overcome by selecting a higher sampling rate at receiver so that the signal can be received at large number of polarization angles and more choices for polarization selection are available to detect the message signal.

Higher order modulations also add complexity to transmitter and receiver architecture. The structure of RPW transmitter is complex because two separate and non-identical PSK modulators are used [24] unlike PD that employs a single modulator. Secondly, as discussed above, channel models used for PD have limitations in terms of complexity and modeling inaccuracies. Therefore, the conventional models for PD systems must be avoided, as this will further increase the complexity in simulation and performance evaluation. The quaternion model can significantly reduce the complexity in performance analysis and simulation of RPW communication. Therefore, this paper has made the following contributions to improve RPW system:

1. Rotating Polarization Multiple Phase Shift Keying (RP-MPSK) modulation is proposed.
2. Novel quaternion model for RPW communication (Q-RPW) is proposed. The complexity of channel model is halved as compared to classical PD model by using four real random numbers instead of eight for channel modeling. The model is applied to RP-MPSK modulation for BER performance evaluation over Rayleigh fading and interference.
3. Receiver sampling rates for higher order modulations are recommended to make their BER performance compatible with RP-BPSK.

The remaining parts of this paper are organized as follows: Section 2 shows improvement of RPW from RP-BPSK to RP-QPSK and then generalization to RP-MPSK. Q-RPW is proposed in Section 3 and mathematical treatment is presented. BER performance of RP-MPSK is evaluated by simulating the proposed model in Section 4. RP-MPSK modulation is also compared with leading LPWAN modulations. The section also investigates the effect of increase in sampling rate on RP-MPSK to maintain the error performance. Section 5 concludes the contributions and findings of the paper.

The following notations have been used in this article: $\omega_{r}$: frequency of polarization rotation, or frequency of modulation (rad/s); $f_{r}$: frequency of modulation (Hz); $\omega_{c}$: carrier frequency (rad/s); $f_{c}$: carrier frequency (Hz); $T_{r}$: symbol period (s); $h_{t}\left(t\right)$: baseband signal to be transmitted through horizontal polarized antenna; $v_{t}\left(t\right)$: baseband signal to be transmitted through vertically polarized antenna; $E_{b}$: energy per bit; $E_{s}$: energy per symbol; M: order of modulation; k: number of bits in a symbol, $k=\log_{2}M$; m: an integer that limits the number of symbols in *M*th order modulation (m=0,1,2,…,M−1); $s_{m}$: *m*th symbol of *M*th order modulation; $x_{e}$: even bit stream; $x_{o}$: odd bit stream; $a_{x}$: unit vector of *x*-axis; $a_{y}$: unit vector of *y*-axis; β: phase constant; z: displacement in the direction of *z*-axis; *n*: number referring a multipath component; $\phi_{n}$: polarization angle of the *n*th multipath component; $\theta_{n}$: phase of the nth multipath component; $a_{n}$: magnitude of the *n*th multipath component received on horizontal polarized antenna; $b_{n}$: magnitude of the nth multipath component received on vertical polarized antenna; $r_{h}\left(t\right)$: signal received on horizontally polarized antenna; $r_{v}\left(t\right)$: signal received on vertically polarized antenna; $n_{h}$: AWGN component of $r_{h}\left(t\right)$; $n_{v}$: AWGN component of $r_{v}\left(t\right)$; $h_{r}\left(t\right)$: baseband signal obtained from $r_{h}\left(t\right)$; $v_{r}\left(t\right)$: baseband signal obtained from $r_{v}\left(t\right)$; $f_{s}$: sampling frequency; $T_{s}$: sampling interval; $N_{p}$: number of samples per $T_{r}$; *p*: an integer that limits the number of samples ($p=0,1,2,3,\ldots ,N_{p}$); $h_{p}$: demodulated sequence obtained from $h_{r}\left(t\right)$; $v_{p}$: demodulated sequency obtained from $v_{r}\left(t\right)$; $r_{p}$: sequence obtained after combining;$\;{\hat{s}}_{m}$: estimated $s_{m}$; ${\hat{s}}_{m,p}$: estimated *p*th replica of $s_{m}$: $q_{m}$: quaternion symbol corresponding to $s_{m}$; H: quaternion channel; $q_{m,\ell }$: ℓth replica of $q_{m}$ (ℓ=p, $N_{p}=N_{\ell }$); ${\hat{q}}_{m}$: estimated $q_{m}$;$\;{\hat{q}}_{m,\ell ,}$: estimated $q_{m,\ell }$; w: quaternion AWGN.

## 2. Materials and Methods

Previous works on RPW covered only RP-BPSK and RP-QPSK modulation [24]. RP-BPSK was the first modulation scheme used in RPW when it was invented. In this method, a binary data symbol $s_{m}$ simultaneously modulates two orthogonal baseband sinusoids of low frequency $\omega_{r}=2\pi f_{r}=2\pi /T_{r}$ as:

$$\begin{array}{cc} \begin{array}{c} h_{t}\left(t\right)=\pm \sqrt{2E_{b}/T_{r}}\cos\left(\omega_{r}t\right)\\ v_{t}\left(t\right)=\pm \sqrt{2E_{b}/T_{r}}\sin\left(\omega_{r}t\right) \end{array}\} & m=0,1 \end{array}$$

These signals further modulate a carrier of high frequency $\omega_{c}=2\pi f_{c}$ for horizontal and vertical polarized transmissions through DP antenna, respectively. By transmitting the two baseband signals having a phase difference of π/2 along two orthogonal polarizations, the polarization of the resultant electromagnetic signal is made to rotate at frequency $\omega_{r}$, hence called RPW. In analogy with CP, we call this RPW as Right-Hand RPW (RHRPW) (Figure 1). On the receiver side, RPW signal is received by another DP antenna. The signals received on both antennas are sampled at frequency $f_{s}$, that is an integral multiple of $f_{r}$. Each sample obtained in this way on the two antennas is polarized at a different angle and is a replica of the transmitted symbol. For each sample, the signal with higher signal power out of the two polarizations is selected as the desired sample. Each selected sample is then demodulated to recover a replica of the transmitted symbol. If there is a greater number of 1′s than 0′s, the receiver decides in favour of binary 1. A binary 0 is decided otherwise.

**Figure 2:**
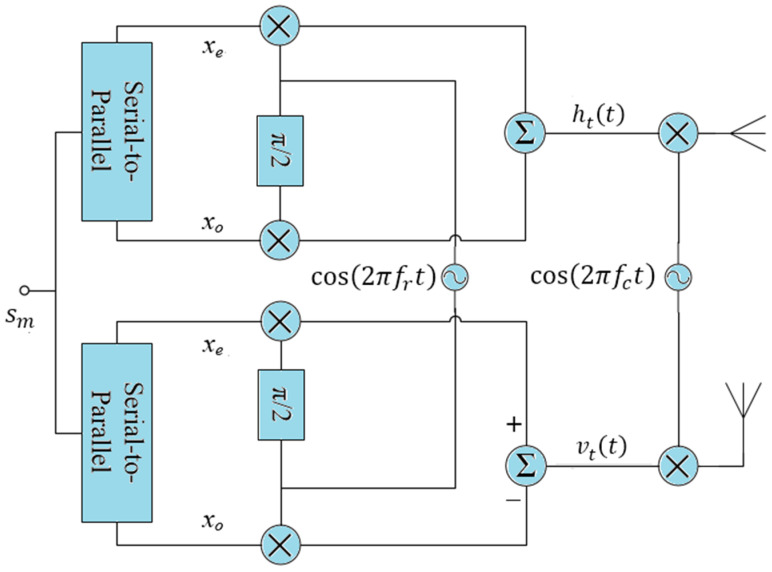
RP-QPSK Transmitter.

From classical theory of modulation, we know that QPSK is spectrally more efficient than BPSK, provides higher data rate and offers BER performance equivalent to BPSK. Motivated by the fact, RP-QPSK was proposed [24]. The main issue in moving to RP-QPSK was that RP-BPSK has already used two quadrature baseband carriers. Using the same carriers to send QPSK symbol as well as two orthogonal polarized signals with a phase difference of 90° at the same time cannot be realized. Also, the two QPSK transmitters should operate on the same baseband frequency to generate RPW. This problem was solved by manipulating the linear combination of the orthogonal basis functions (Figure 2). A symbol $s_{m}\in \left\{s_{0},s_{1},s_{2},s_{3}\right\}$ with energy $E_{s}$ is demultiplexed into even bit $x_{e}$ and odd bit $x_{o}$. The horizontal and the vertical polarized signals for RP-QPSK are described by the following equations:

(1) $$\begin{array}{c} h_{t}\left(t\right)=x_{e}\psi_{1}\left(t\right)+x_{o}\psi_{2}\left(t\right)\\ v_{t}\left(t\right)=x_{e}\psi_{2}\left(t\right)-x_{o}\psi_{1}\left(t\right) \end{array}$$

Here, $\psi_{1}\left(t\right)$ are $\psi_{2}\left(t\right)$ are the orthogonal basis functions defined by

$$\begin{array}{cc} \psi_{1}\left(t\right)=\sqrt{2/T_{r}}\cos\left(\omega_{r}t\right) & \psi_{2}\left(t\right)=\sqrt{2/T_{r}}\sin\left(\omega_{r}t\right) \end{array}$$

Note that even and odd bits have swapped positions in the signals to be transmitted through horizontal and vertical polarizations. Another difference is the addition of the two terms in the signal for horizontal polarization and subtraction in the signal for vertical polarization. RP-QPSK modulation can now be expressed in more general form as below:

(2) $$\begin{array}{cc} \begin{array}{c} h_{t}\left(t\right)=\sqrt{\frac{2E_{s}}{T_{r}}}\cos\left(\omega_{r}t+\left(2m+1\right)\frac{\pi }{4}\right)\\ v_{t}\left(t\right)=\sqrt{\frac{2E_{s}}{T_{r}}}\sin\left(\omega_{r}t+\left(2m+1\right)\frac{\pi }{4}\right) \end{array}\} & m\in 0,1,2,3 \end{array}$$

The demodulation process of RP-BPSK is adopted to recover transmitted symbol. Further details and the demodulation procedure will be covered in RP-MPSK modulation.

### 2.1. Rotating Polarization-MPSK (RP-MPSK) Modulation

BER performance of RPW remains unaffected if RP-QPSK is used instead of RP-BPSK, but more transmitted power is required because two quadrature carriers are used [24]. To further improve the data rate and the energy efficiency, RP-MPSK modulation is proposed. The data rate increases at the cost of BER performance. The problem can be mitigated if RPW receiver samples the received signal at higher sampling rates to combat BER degradation.

Let the order of modulation be $M=2^{k}$ when there are $k=\log_{2}M$ bits per symbol $s_{m}\in \left\{s_{0},s_{1},s_{2},\ldots ,s_{m-1}\right\}$. The step-by-step process from modulation to detection is given below.

#### 2.1.1. RP-MPSK Modulation

**Figure 3:**
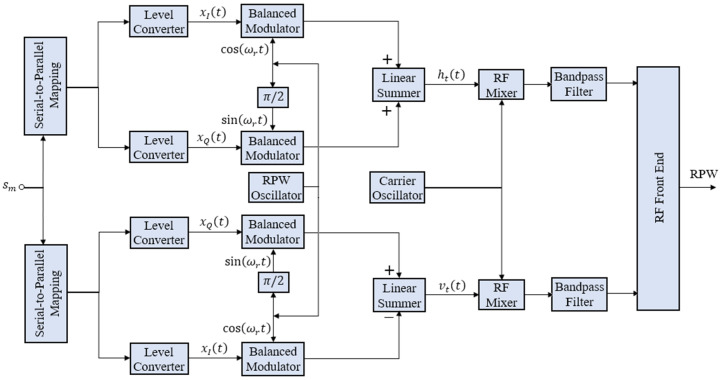
Block Diagram of RP-MPSK Transmitter.

RPW is made feasible by equipping the transmitter in Figure 3 with two baseband PSK modulators of frequency $\left(\omega_{r}=2\pi f_{r}=2\pi /T_{r}\right)$, but orthogonal to each other for horizontal and vertical polarizations. A data symbol $s_{m}$ is simultaneously input to both modulators to generate $h_{t}\left(t\right)$ and $v_{t}\left(t\right)$, where the subscript *t* corresponds to transmitted signal.

(3) $$\begin{array}{cc} \begin{array}{c} h_{t}\left(t\right)=\sqrt{\frac{2E_{s}}{T_{r}}}\cos\left(\omega_{r}t+\frac{2m\pi }{M}\right)\\ v_{t}\left(t\right)=\sqrt{\frac{2E_{s}}{T_{r}}}\sin\left(\omega_{r}t+\frac{2m\pi }{M}\right) \end{array}\} & m\in \left\{0,1,2,\ldots ,M-1\right\} \end{array}$$

#### 2.1.2. RPW Transmission and Reception

The baseband modulated signals are upconverted to RF carrier $\left(\omega_{c}=2\pi f_{c}\right)$ of ISM band. The electromagnetic signal is mathematically described as

(4) $$E\left(z;t\right)=\left[h_{t}\left(t\right)a_{x}+v_{t}\left(t\right)a_{y}\right]\cos\left(\omega_{c}t-\beta z\right).$$

**Figure 4:**
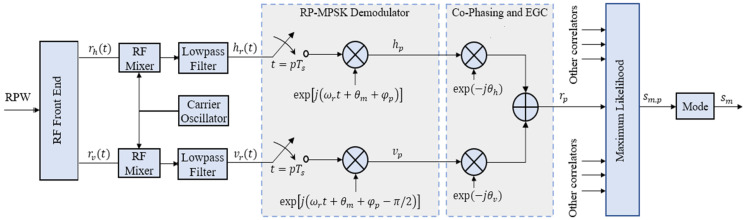
Coherent RP-MPSK Receiver with Equal Gain Combining (EGC).

RP-MPSK transmitter is like RP-QPSK transmitter in Figure 1. However, the serial to parallel logic varies with higher order of modulation. A block diagram of the digital implementation of RP-MPSK receiver is shown in Figure 4.

The signals received on the two elements of dual-polarized antenna are sufficiently degraded due to scattering, multipath regular and irregular reflections:

(5) $$\begin{array}{c} r_{h}\left(t\right)=h_{t}\left(t\right)\underset{n}{\sum }a_{n}\cos\varphi_{n}\cos\left(\omega_{c}t+\theta_{n}\right)+n_{h}\\ r_{v}\left(t\right)=v_{t}\left(t\right)\underset{n}{\sum }b_{n}\sin\varphi_{n}\cos\left(\omega_{c}t+\theta_{n}\right)+n_{v} \end{array}$$

These signals are down-converted to baseband signals $h_{r}\left(t\right)$ and $v_{r}\left(t\right)$, respectively.

#### 2.1.3. RP-MPSK Demodulation

Received baseband signals are sampled at frequency $f_{s}=1/T_{s}=N_{p}f_{r}\ll f_{c},N_{p}\in Z$. Each sample is then coherently demodulated as below:

(6) $$\begin{array}{cc} \begin{array}{c} h_{p}=h_{r}\left(pT_{s}\right)\cos\left(\omega_{r}pT_{s}+2p\pi /N_{p}\right)\\ v_{p}=v_{r}\left(pT_{s}\right)\sin\left(\omega_{r}pT_{s}+2p\pi /N_{p}\right) \end{array}\} & p\in \left\{0,1,2,\ldots ,N_{p}-1\right\} \end{array}$$

#### 2.1.4. Combining

The previous works on RPW used selection combining (SC). We suggest using Equal Gain Combining (EGC) since it performs better than SC with constant-power envelope (CPE) modulations like MPSK. Both are presented here:

(7) $$\mathrm{SC}:\;r_{p}∶=\{\begin{array}{cc} h_{p} & {\left|h_{p}\right|}^{2}\geq {\left|v_{p}\right|}^{2}\\ v_{p} & \mathrm{otherwise} \end{array}$$

(8) $$\mathrm{EGC}:\;r_{p}∶=\frac{1}{2}\left(h_{p}+v_{p}\right)$$

Here, equal noise power spectral density is assumed for horizontal and vertical polarized signals in (8). The signals are co-phased before EGC. The structure of coherent RP-MPSK receiver with EGC is shown in Figure 4.

Maximum likelihood detection (MLD) is performed on each $r_{p}$ resulting in $N_{p}$ replicas of the transmitted symbol $s_{m}$, denoted by ${\hat{s}}_{m,p}$:

(9) $$\begin{array}{cc} {\hat{s}}_{m,p}≜\arg\underset{m}{\max}ℜ\left\{r_{p}\cdot s_{m}^{*}\right\} & p\in \left\{0,1,2,\ldots ,N_{p}-1\right\} \end{array}$$

Decision on the symbol estimate ${\hat{s}}_{m}$ is made in favour of ${\hat{s}}_{m,p}$ that has most frequently occurred:

(10) $${\hat{s}}_{m}≜\mathrm{mode}\left(\left\{{\hat{s}}_{m,p}\right\}\right)$$

### 2.2. Quaternion Model for RPW Communication (Q-RPW)

Q-RPW model is motivated by the polarization of electromagnetic waves arriving at a receive antenna in multipath environment, given below as [20]:

(11) $$\begin{array}{cc} r\left(t\right) & =\underset{n}{\sum }c_{n}e^{j\varphi_{n}}\cos\left(\omega_{c}t+\theta_{n}\right)\\ & =\underset{n}{\sum }c_{n}\left[\cos\varphi_{n}\cos\theta_{n}\cos\left(\omega_{c}t\right)+\cos\varphi_{n}\sin\theta_{n}\sin\left(\omega_{c}t\right)+j\sin\varphi_{n}\cos\theta_{n}\left(\omega_{c}t\right)+j\sin\varphi_{n}\sin\theta_{n}\sin\left(\omega_{c}t\right)\right] \end{array}$$

Since the change in polarization angle is independent of the phase of transmitted signal, the trigonometric coefficients of the carrier in the four terms of (11) are independent. That is exactly the case in a quaternion variable. Hence RPW can be expressed in quaternion form. In this section, new Q-RPW model for RPW communication is presented.

The proposed model is based on quaternion representation of dual-polarized systems [17,18]. Quaternion symbols for a dual-polarized system are represented as:

(12) $$q_{m}=s_{H}+s_{V}j$$

Equation (12) states that two complex symbols $s_{H}=a_{H}+ib_{H}$ and $s_{V}=a_{V}+ib_{V}$ $\left(a_{H},b_{H},a_{V},b_{V}\in R\right)$ are transmitted through horizontal and vertical polarized elements of a dual-polarized antenna.

To represent RP-MPSK symbols, we change (12) in accordance with (3). Since the baseband modulation frequencies have a phase difference of π/2, we deduce that for RP-MPSK, $s_{V}=s_{H}e^{-i\pi /2}$. We use (12) to define quaternion RP-MPSK symbol as:

(13) $$q_{m}≜a+bi-bj+ak$$

Here, $s_{m}=s_{H}=a+ib$ is the conventional MPSK symbol. The imaginary numbers i,j,k are defined by:

$$ij=-ji=k,jk=-kj=i,ki=-ik=j,i^{2}=j^{2}=k^{2}=ijk=-1$$

A rotation of $q_{m}$ in the polarization domain by an angle $\varphi_{\ell }$ can be represented as $q_{m,\ell }=q_{m}e^{-j\varphi_{\ell }\;}$ [17]. For RPW transmission, we rotate $q_{m}$ by $N_{\ell }$ polarization angles to generate the sequence $\left\{q_{m,\ell }\right\}$, such that:

(14) $$\begin{array}{cc} q_{m,\ell }=q_{m}e^{-j2\ell \pi /N_{\ell }} & \ell =0,1,2,\ldots ,N_{\ell }-1 \end{array}$$

From implementation perspective, this is equivalent to interpolating the transmitted symbol stream $\left\{q_{m}\right\}$ by a factor $N_{\ell }$. The digital quaternion stream $\left\{q_{m,\ell }\right\}$ is up-converted to analog RPW signal for RF transmission through dual polarized antenna.

The RF signal arrived at dual-polarized receive antenna is down-converted to analog baseband signal and sampled at a frequency $f_{s}=N_{p}f_{r}$ specified in previous section. This is important to emphasize that $N_{\ell }$ and $N_{p}$ can be chosen independent of each other; however, for convenience, we take $N_{\ell }=N_{p}$. The sampled discrete signal is converted to baseband quaternion stream $r_{m,\ell }$ that can be represented in the form of (12):

(15) $$r_{m,\ell }=q_{m,\ell }H+w$$

Here H represents the quaternion channel and w represents AWGN at receiver. The channel is described by the following equation:

(16) $$H≜h_{o}+h_{i}i+h_{j}j+h_{k}k$$

The coefficients $h_{0},h_{i},h_{j},h_{k}∼N_{R}\left(0,\sigma_{h}^{2}\right)$ are real i.i.d. normal random variables. We can also express the channel in a more compact form as $H∼N_{H}\left(0,\sigma_{H}^{2}\right)$ where H is the quaternion or the Hamilton space. Similarly, the noise can be expressed as $w\sim N_{H}\left(0,\sigma_{w}^{2}\right)$.

The model of the received signal is related to conventional model of PD system, starting from the following expression consistent with (12):

(17) $$r_{m,\ell }=r_{H}+r_{V}j$$

Here $r_{H}$ is the signal received by the horizontal polarized antenna and $r_{V}$ is the signal received by the vertical polarized antenna. If $h_{HH}$, $h_{VV}$ were the complex channel coefficients of copolarized links and $h_{VH}$, $h_{HV}$ the complex channel coefficients of cross-polarized links, we can write [17]

(18) $$r_{H}=s_{m,\ell }\left(h_{HH}+e^{-i\pi /2}h_{VH}\right)\;r_{V}=s_{m,\ell }\left(h_{HV}+e^{-i\pi /2}h_{VV}\right)$$

where $s_{m,\ell }$ is obtained by a phase shift $\varphi_{\ell }$ in $s_{m}$. It was shown in [17] that the channel coefficients of quaternion model and the conventional model are related by:

$$\begin{array}{ll} h_{o}=ℜ\left\{h_{HH}\right\}=ℜ\left\{h_{VV}\right\} & h_{i}=ℑ\left\{h_{HH}\right\}=-ℑ\left\{h_{VV}\right\}\\ h_{j}=-ℜ\left\{h_{VH}\right\}=ℜ\left\{h_{HV}\right\} & h_{k}=ℑ\left\{h_{VH}\right\}=ℑ\left\{h_{HV}\right\} \end{array}$$

This is the significant advantage of quaternion model. The number of real random variable that model the channel are halved by using quaternion model [17]. To recover the transmitted sequence $\left\{q_{m,\ell }\right\}$, MLD is performed on each $r_{m,\ell }$ by evaluating the following expression [17,18]:

(19) $${\hat{q}}_{m,\ell }≜\arg\underset{m}{\min}{\left||r_{m,\ell }-q_{m,\ell }H|\right|}^{2}\;=\arg\underset{m}{\min}\left[\left(r_{m,\ell }-q_{m,\ell }H\right){\left(r_{m,\ell }-q_{m,\ell }H\right)}^{Q}\right]$$

Here, ${\left(\cdot \right)}^{Q}$ is the quaternion conjugate. Finally, to recover the transmitted symbol $q_{m}$, the estimate ${\hat{q}}_{m,\ell }$ that has most frequently occurred is selected:

(20) $${\hat{q}}_{m}≜\mathrm{mode}\left(\left\{{\hat{q}}_{m,\ell }\right\}\right)$$

**Figure 5:**
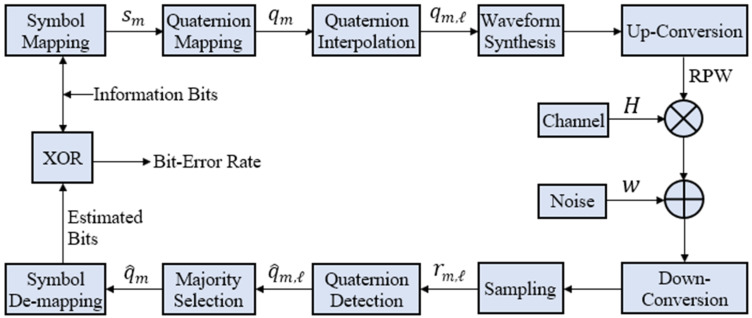
Performance evaluation of RP-MPSK modulation and demodulation using Q-RPW Model.

Hence, the RP-MPSK transmitted symbol stream $\left\{q_{m}\right\}$ has been recovered as $\left\{{\hat{q}}_{m}\right\}$ using quaternion model for RPW. A block diagram for implementation of the quaternion model for RP-MPSK modulation is in Figure 5.

## 3. Results

Q-RPW model has simplified the simulation and performance evaluation of RPW system. As mentioned in previous section, it has halved the number of real random gaussian variables required to model the channel [17]. Since each symbol is received $N_{p}$ times in RPW, this reduction translates to a high computational efficiency. Q-RPW has also improved the performance of RPW receiver by combining the signals received from two branches using quaternions. This method is superior to both SC and EGC because the orthogonality of received polarizations is conserved.

Figure 5 shows the procedure adopted for simulation of quaternion model of RPW and BER evaluation of RP-MPSK. MATLAB simulation is performed, and the simulation parameters are shown in Table 1. The simulation results are divided into four parts: BER performance of RP-MPSK modulation, comparison of BER of RP-MPSK with other LPWAN modulation schemes, BER evaluation for recommended sampling rates (value of $N_{p}$) at receiver, and BER performance under interference and multipath conditions.

### 3.1. Performance of RP-MPSK Modulation

We considered RP-BPSK, RP-QPSK, RP-8-PSK, and RP-16-PSK. Going beyond 16-PSK results in closely spaced constellation points. The angular spacing for 16-PSK modulation is 22.5°, therefore a maximum phase error of 11.25° in a symbol is permissible on the receiver to correctly detect the symbol. In case of 32-PSK, the margin for random phase error is slightly higher than 5°. Heavy multipath environments, and the errors rendered by PLL are prone to more errors because of small allowable phase error. The performance of further higher orders in MPSK is even worse [25].

**Figure 6:**
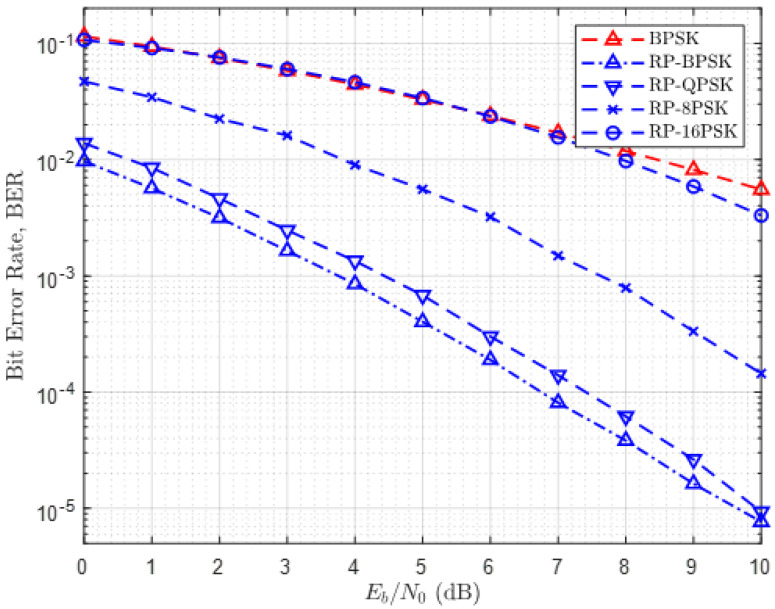
BER Performance of RP-BPSK, RP-QPSK, RP-8-PSK and RP-MPSK at $N_{p}=3$.

In our case, to demonstrate the superior performance of RP-MPSK, BERs of RP-BPSK, RP-QPSK, RP-8-PSK, and RP-16-PSK are compared with theoretical BER of BPSK where second-order diversity is exploited as a function of $\alpha =E_{b}/N_{o}$ (Figure 6). The rationale for this comparison is that RP-MPSK also exploits second-order diversity. Sampling rate of only three times the RPW frequency $\left(N_{p}=3\right)$ is chosen. This choice of sampling rate is quite logical for preliminary validation. Since the final decision on symbol estimation in RP-MPSK is based on the most frequently occurring symbol, RP-MPSK cannot perform correct decision if $N_{p}=2$ is used. Significant diversity gain is achieved using RP-MPSK instead of BPSK. The BERs of RP-BPSK, RP-QPSK, and RP-8-PSK are remarkably improved compared to BPSK. In case of RP-BPSK and RP-QPSK, an ample improvement of about 8 dB in α is observed to achieve a small BER of 1%. Following this trend, an improvement of 10 dB or more in α is anticipated to achieve BER of 0.1%. It is indeed interesting to note that RP-16-PSK performs equivalent to BPSK with a sampling rate slightly above the Nyquist rate $\left(N_{p}=3\right)$. This improvement can be attributed to the fact that the three samples thus obtained come from three different polarizations. A few more common observations can also be made. For example, the performance of RP-BPSK and RP-QPSK is equivalent, and they offer the lowest BER. RP-8-PSK outperforms RP-16-PSK.

### 3.2. Comparison of RP-MPSK with Other LPWAN Modulations

**Figure 7:**
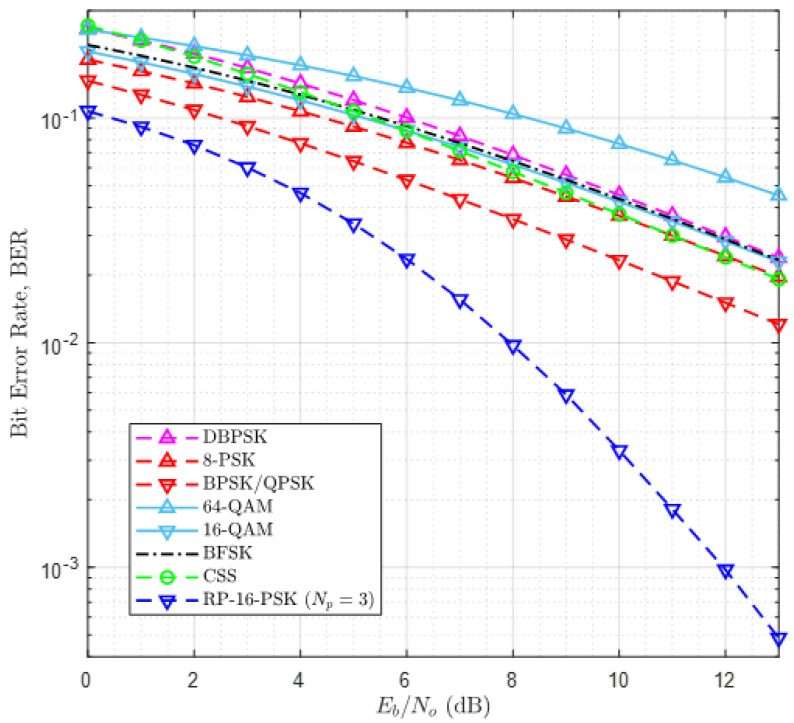
Comparison of RP-MPSK with other LPWAN modulations.

RPW operates on sub-gigahertz and other lower ISM bands. ISM band along with a few parts of licensed spectrum, are the bands of interest for most of the LPWANs that have gained global attention. Therefore, we have compared the performance of RP-MPSK with other LPWAN modulations. Table 2 lists different uplink modulation schemes and LPWANs in which they are used [5]. Figure 7 shows a comparison of their BER performance. 64-QAM has the highest degradation in BER while RP-16-PSK with $N_{p}=3$ offers the best BER. It can be observed that BERs of 8-PSK, 16-QAM, BFSK and CSS are similar therefore the curves overlap. It is important to mention here that BER curve of CSS holds for all values of SF when plotted against α [26]. BPSK and QPSK outperform all other modulations except RP-16-PSK. Hence, we infer that RPW with RP-MPSK modulation is a highly reliable LPWAN solution, and therefore it can be used for industrial communication. The results also show that it offers a higher energy efficiency than other LPWAN technologies. RPW with RP-16-PSK is also a good trade-off between high data rate and high energy efficiency, as a reliable high data rate up to 2 Mbps is realizable.

### 3.3. Recommended Sampling Rates to Compensate Performance Degradation of RP-MPSK

**Figure 8:**
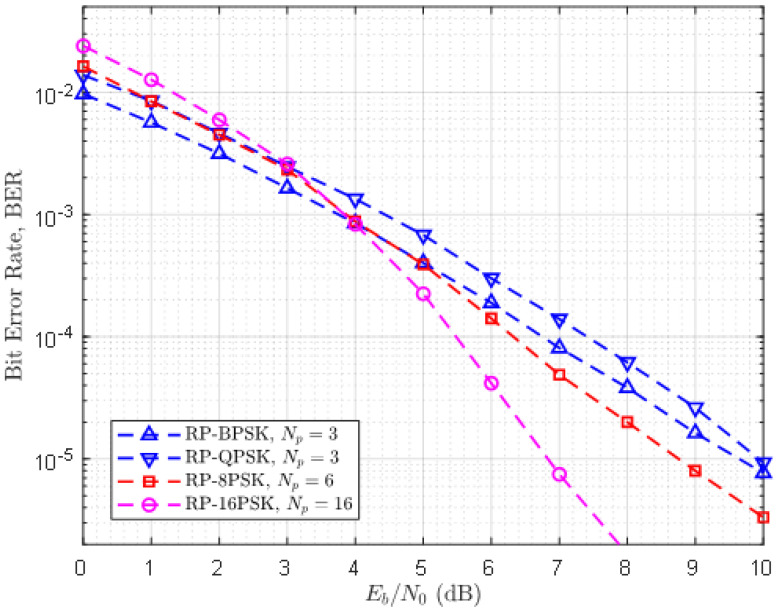
Recommended sampling rates for RP-8-PSK and RP-16-PSK for compatibility with RP-BPSK/RP-QPSK.

The trade-off between data rate and BER is the key design aspect in PHY design as highlighted in Section 1. The aim of simulation in this part was to identify the best value of $N_{p}$ for RP-8-PSK and RP-16-PSK that can deliver the same BER as that of RP-BPSK and RP-QPSK against the same values of α. For consistency with previous results, we take $N_{p}=3$ as the reference value for RP-BPSK and RP-QPSK. To observe how the error-performance of RP-MPSK depends on $N_{p}$, BER of RP-8-PSK and RP-16-PSK for several values of $N_{p}$ was investigated. Figure 8 shows that the BER of RP-8-PSK and RP-16-PSK when their BER closely resembled the BER of RP-BPSK and RP-QPSK. The corresponding values of $N_{p}$ are 6 and 16 for RP-8-PSK and RP-16-PSK, respectively. BER for other values of $N_{p}$ have been omitted to confine our discussion according to the context.

Another interesting finding is extracted from this simulation. For the values of α exceeding 4 dB, the performance of RP-8-PSK and RP-16-PSK supercedes the performance of both RP-BPSK and RP-QPSK. This is promising yet a rational result. The performance of MFSK improves with higher order of modulation. A similar argument can be made about RP-MPSK. Since RP-MPSK relies on the value of $N_{p}$ to enhance the reliability, arbitrary higher sampling rate has shown the improved performance of RP-8-PSK and RP-16-PSK. However, unlike MFSK, channel bandwidth is not increased with higher order modulations. Thus, we deduce that RP-MPSK has a higher spectral efficiency than MFSK, while it can potentially approach the BER of MFSK.

### 3.4. BER Performance under Interference and Multipath Conditions

The performance of communication systems in general is limited by interference. In case of MPSK modulations, it is of particular importance. Therefore, the BER performance of RP-MPSK is investigated under multipath and interference conditions occurring at the same time. The case of co-channel interference from single user is considered [27] assuming matched central frequency. Equation (15) can be rewritten for this case as:

(21) $$r_{m,\ell }=q_{m,\ell }H+\mu q_{\left(m,\ell \right)i}H_{i}+w$$

**Figure 9:**
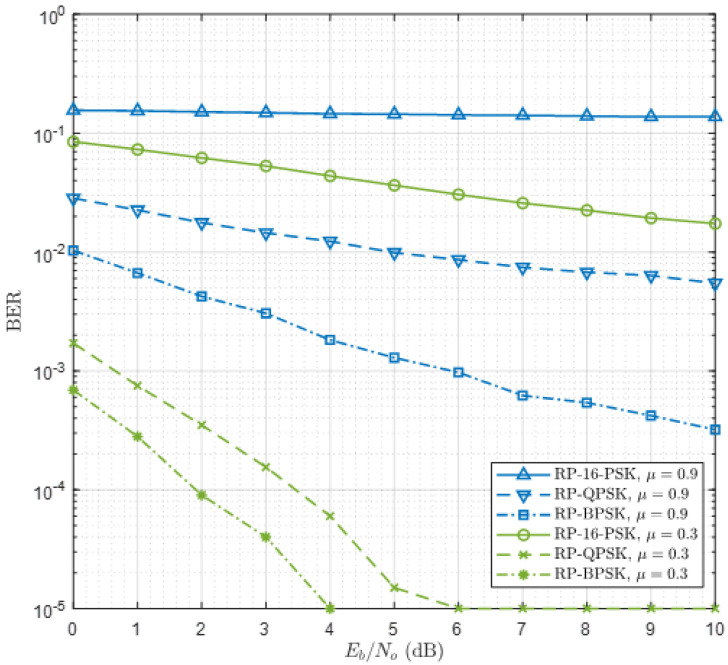
Performance of RP-MPSK under multipath and Interference for $N_{p}=7$.

Here, $q_{\left(m,\ell \right)i}$ is the replica of the symbol transmitted by the interfering user, $H_{i}$ is the quaternion fading coefficient of the interfering user, and *μ* is the relative level of interference. Two interference cases are considered: a low interference (*μ* = 0.3) and a high interference (*μ* = 0.9). In Figure 9, BER curves of RP-MPSK are obtained for M=2, 4, 16 and $N_{p}=7$. Since interference always degrades the BER performance, a higher value of $N_{p}$ compared with Figure 6 is selected. The result shows that for low to moderate BER, RP-BPSK and RP-QPSK must be preferred. According to Figure 8, RP-16-PSK can also be used provided a high value of $N_{p}$ is selected. The same argument is also valid for RP-8-PSK though its results are not shown. Comparing Figure 9 with Figure 6 reveals that the performance of RP-BPSK ($N_{p}=7$) with interference and multipath is consistent with the performance of RP-8-PSK ($N_{p}=3$) with multipath only, for low values of α.

## 4. Discussion

The performance of RP-MPSK can be discussed in three contexts: data rate, link reliability, and energy efficiency. With the proposed RP-MPSK modulation for RPW, a data rate of up to 2 Mbps is realizable for M=16. This is comparable to the data rate of LTE-M. With RP-QPSK, a data rate higher than NB-IoT can be achieved [24]. With the lowest order of modulation, i.e., RP-BPSK, the data rate of 125 kbps is higher than most of the existing LPWANs. The high data rate also translates to the spectral efficiency because the same bandwidth is made capable to transmit higher data rate using RP-MPSK.

The context of reliability can be more clearly stated in terms of diversity gain. Figure 6 exhibits a large diversity gain of RP-BPSK over simple BPSK that exploits second-order diversity. According to Figure 7, RP-16-PSK with $N_{p}=3$ attains significant diversity gain over 8-PSK and 16-QAM without diversity. Since LPWANs do not employ spatial diversity, it is not considered for this comparison. Another interesting aspect of the reliability of RP-MPSK is its flexibility to achieve desired BER with any value of *M*. Generally higher order PSK modulations degrade the error performance of a communication system. But in RP-MPSK the value of $N_{p}$ can be increased to fetch the required BER. However, this is limited by hardware specification of the receiver.

The last and the most important context is the energy efficiency of RP-MPSK. This can be better explained from two aspects. In the simplest way, the energy efficiency of RP-MPSK modulation comes from the gain in $\alpha =E_{b}/N_{o}$ as shown in Figure 6 and Figure 7. The desired BER can be achieved at a much lower value of α compared with other modulation methods and the most reliable BPSK modulation with second order diversity is not an exception. Secondly, based on our argument in Section 1, use of RP-MPSK encapsulates more amount of data by the same amount of energy without an increase in the symbol duration. Since a communication system consumes highest energy while it is transmitting and most of the power is consumed by the power amplifier, an improvement of as low as 5 dB in α for every bit transmitted using RP-MPSK makes a significant contribution to cut down overall power consumption of RPW nodes. Improvement in α can also be viewed as improvement in SNR of the transmitted signal. With RP-MPSK, the required BER can be achieved at a substantially lower SNR as compared to other modulations considered in this article. Therefore, the sensitivity of the receiver is also improved offering more fade margin.

We now summarize the error performance of RPW with the proposed MPSK modulation. In terms of BER, RPW performs better than other LPWAN modulation schemes. The receiver sensitivity and BER rate can be controlled at RP-MPSK receiver by adjusting the sampling rate according to the design requirements of the system.

RPW has a huge potential to grow and mature as a leading LPWAN with the proposed modulation scheme. The data rate obtained by RP-MPSK is sufficient for most of the M2M applications. However, Rotating Polarization MQAM (RP-MQAM) can be proposed and employed for further increment in data rate. RP-MPSK can also be made to compete WiFi-HaLow if RP-MPSK and RP-MQAM modulations exploit orthogonal frequency division multiplexing (OFDM). Maximal Ratio Combining (MRC) can be considered in addition to SC and EGC using classical model of PD. Error correction codes can be implemented to achieve ultra-high reliability. The results indicate that RP-MPSK offers higher sensitivity. Therefore, link budget analysis of RPW system should be investigated for comparison with other LPWANs to estimate the achievable range [28]. Experiments should be performed in real industrial environment for performance evaluation of RP-MPSK. Limitation on maximum sampling rate should be investigated by prototyping and SDR based implementation. Research should be carried out to propose methods and algorithms for upper layer design goals and objectives. Existing protocols can also be investigated to devise a complete protocol stack for RPW communication. Sensor-fault detection is a considerable issue in the deployment of digital twins in Industry 4.0 [29]. RPW can be investigated to solve this problem. The challenges of link quality, noise and interference, and environmental impacts in wireless sensor networks can also be addressed by employing RPW [30]. Use of RP-MPSK for non-industrial applications such as mobile communication is another potential research direction. RPW also has a great potential to solve the problem of reliable broadband connection in remote and rural areas. It can provide a reliable satellite link by mitigating atmospheric perturbations. Another interesting application is non-destructive testing of orthotropic materials [31]. In short, since RPW is a nascent wireless communication technology, it has a vast room for researchers to explore on various levels and in various fields.

## 5. Conclusions

RP-MPSK modulation is proposed for Industry 4.0 and M2M communication that offers a data rate up to 2 Mbps. Simulation results have shown that RP-MPSK is a robust and flexible modulation technique that can deal with various design goals for LPWANs. High reliability due to superior error performance, greater power efficiency due to increased sensitivity, and efficient use of bandwidth are the main advantages of this modulation. To simplify the complex RPW channel, Q-RPW model is proposed that also improves the quality of reception with quaternion combining. Q-RPW model can also reduce the complexity of stochastic analysis to obtain a few important performance metrics such as BER over fading channels. Channel estimation technique for RPW communication system can also be simplified with Q-RPW. Future works include stochastic analysis of RPW with RP-MPSK modulation, a rigorous comparison of LoRa and RPW physical layers, MAC layer implementation, and development of RPW transceiver prototype employing RP-MSPK.

## Figures and Tables

**Table 1:** RP-MPSK Simulation Parameters.

Parameter	Value
$\mathrm{No}.\;\mathrm{of}\;\mathrm{Symbols},\;N_{s}$	100,000
$\mathrm{No}.\;\mathrm{of}\;\mathrm{Monte}\;\mathrm{Carlo}\;\mathrm{Trials},\;N_{t}$	25, 100
$\mathrm{No}.\;\mathrm{of}\;\mathrm{Samples}\;\mathrm{per}\;\mathrm{symbol}\;\mathrm{period},\;N_{p}$	3, 6, 7, 16
Order of Modulation, M	2, 4, 8, 16
$\mathrm{Bit}\;\mathrm{Energy}\;\mathrm{to}\;\mathrm{Noise}\;\mathrm{Ratio},\;E_{b}/N_{o}$	0–10 dB
Interference level, μ	0.3 (low), 0.9 (high)

**Table 2:** LPWAN Modulation Schemes.

Modulation	LPWAN
DBPSK	SigFox, DASH7
8-PSK	EC-GSM
BPSK, QPSK	NB-IoT, LTE-M
16-QAM, 64-QAM	LTE-M, WiFi-HaLow
CSS	LoRa
BFSK	LoRa
RP-16-PSK	RPW

## Data Availability

Not applicable.
